# Targeting tissue-specific metabolic signaling pathways in aging: the promise and limitations

**DOI:** 10.1007/s13238-013-0002-3

**Published:** 2014-01-29

**Authors:** Fang Hu, Feng Liu

**Affiliations:** Metabolic Syndrome Research Center, The Second Xiangya Hospital of Central South University, Changsha, 410011 China

**Keywords:** aging, metabolic disease, insulin, mTOR, caloric restriction

## Abstract

It has been well established that most of the age-related diseases such as insulin resistance, type 2 diabetes, hypertension, cardiovascular disease, osteoporosis, and atherosclerosis are all closely related to metabolic dysfunction. On the other hand, interventions on metabolism such as calorie restriction or genetic manipulations of key metabolic signaling pathways such as the insulin and mTOR signaling pathways slow down the aging process and improve healthy aging. These findings raise an important question as to whether improving energy homeostasis by targeting certain metabolic signaling pathways in specific tissues could be an effective anti-aging strategy. With a more comprehensive understanding of the tissue-specific roles of distinct metabolic signaling pathways controlling energy homeostasis and the cross-talks between these pathways during aging may lead to the development of more effective therapeutic interventions not only for metabolic dysfunction but also for aging.

## Introduction

The normal process of aging is associated with progressive deterioration in both structure and function of various molecular, cellular, and tissue components that can be influenced by both genetic and environmental factors. A number of theories have been proposed to explain the aging progress, such as shortening and/or loss of telomere, accumulation of damaged DNA in cells, and dysfunction of important cellular organelles such as the endoplasmic reticulum (ER) and mitochondria. While it is well established that aging is a major risk factor for the progression of various metabolic diseases such as central obesity, insulin resistance, hypertension and type 2 diabetes, much less is known on the links between aging and these metabolic disorders at the molecular and cellular levels.

A great progress has been made in the past decade on the association between aging and various metabolic diseases. Pharmacological or genetic manipulations of key signaling pathways involved in the regulation of glucose and energy metabolism, such as the insulin and the mammalian target of rapamycin (mTOR) signaling pathways, have been shown to improve health-span and longevity in diverse model organisms such as yeast, worms, flies, and mammals (Kennedy and Kaeberlein, 2009; McCormick et al., 2011; Laplante and Sabatini, 2012). An interesting question remains to be answered is what are the underlying mechanisms by which altering the insulin/insulin-like growth factor 1 (IGF-1) or the mTOR signaling pathway suppresses or delays aging-associated diseases and extends lifespan. There is some evidence suggesting that the maintenance of normal ER and mitochondrial function could be a primary longevity determinant. Consistent with this view, caloric restriction (CR), which is the best known intervention that prolongs lifespan in various organisms (Guarente, 2008; Kenyon, 2010), reduces mTOR and insulin/IGF-1 signaling (Bonawitz et al., 2007; Katic et al., 2007), increases mitochondrial biogenesis and/or respiratory activity (Nisoli et al., 2005; Bishop and Guarente, 2007; Zid et al., 2009), and alleviates ER stress (Tsutsumi et al., 2011). In addition to improved ER and mitochondrial function, autophagy related genes have also been found to be involved in cell survival and longevity in various long-lived mutant nematodes and promote survival in worms and flies exposed to prolonged starvation (Gomez and Clarke, 2007; Juhász et al., 2007). Current evidence shows that autophagy is required for ER stress-associated apoptosis and mitochondrial turnover, thus may mediate the integration of the insulin/IGF-1 and mTOR signaling pathways with other cellular machineries in regulating longevity (Meijer and Codogno, 2009; Vellai, 2009). In addition, CR facilitates the degradation of damaged organelles, DNAs and protein aggregates in cells by induction of autophagy (Bergamini et al., 2003; Kim et al., 2007).

In this review, we have summarized recent progresses on the links between aging and metabolic diseases, focusing on key signaling pathways such as the insulin/IGF-1 and their tissue specific function in aging. We have also discussed several potential cellular mechanisms underlying aging and aging-associated metabolic diseases.

## Metabolic Signaling Pathways

### Insulin/IGF signaling in metabolic regulation and aging

The insulin/IGF-I signaling pathway plays an essential role in the regulation of various cellular activities such as lipid and carbohydrate metabolism, gene expression, and cell differentiation, growth, and survival. It is the first discovered and evolutionarily conserved signaling pathway involved in the determination of lifespan and is probably the best characterized regulator of longevity across species (Kenyon, 2010, 2011).

**Figure 1:**
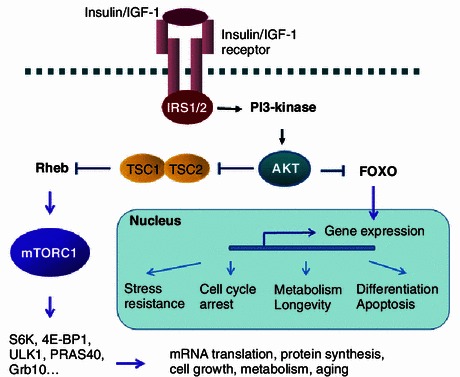
Insulin/IGF-1 signaling and regulation. The binding of insulin or IGF-1 to the membrane receptors leads to the activation of the PI 3-kinase/Akt signaling pathway and subsequent downstream events such as phosphorylation of FOXO1 and activation of the mTORC1 signaling pathway, which regulate many important cellular events such as mRNA translation, protein synthesis, cell cycle progression, metabolism, and aging

Insulin/IGF1 stimulates tyrosine phosphorylation of the insulin receptor (IR) and its substrate (IRS) proteins, IRS1 and IRS2, which, in turn, activate the phosphoinositide 3-kinase (PI3K)/AKT signaling pathway (Fig. 1). Numerous AKT substrates have been identified, including the forkhead box O (FOXO) protein, tuberous sclerosis 2 (TSC2), and many others. Members of the FOXO transcription factor family (FOXO1, FOXO3a, FOXO4, and FOXO6 in mammalians; DAF-16 and DFOXO in *C. elegans* and *Drosophila*, respectively) control the expression of genes involved in the regulation of cell cycle, apoptosis, DNA repair, metabolism, oxidative stress resistance, and aging. The insulin/IGF-1 signaling pathways are highly conserved across species throughout evolution, ranging from worms, flies, rodents to humans, demonstrating the importance of this signaling pathway in the maintenance of normal physiological activities and longevity in these species (van der Horst and Burgering, 2007; Narasimhan et al., 2009).

Suppressing the insulin/IGF-I signaling pathway has been shown to increase lifespan and delay aging process in species ranging from *C. elegans* (Wolkow et al., 2000; Wolff and Dillin, 2006), yeast (Fabrizio et al., 2001), *Drosophila* (Clancy et al., 2001; Tatar et al., 2001; Zhang et al., 2009) to rodents (Holzenberger et al., 2003). In *C. elegans*, loss-of-function mutations of the DAF-1/FOXO upstream kinases such as DAF-2 and aging alteration-1 (age-1) promoted DAF-16/FOXO protein translocation into the nucleus to activate or repress its target genes (Kenyon, 2005; Calnan and Brunet, 2008). Extended longevity is also observed in *C. elegans* with mutations in age-1 and daf-2, which encode the catalytic subunit of the worm PI3K and the insulin/IGF-1 receptor, respectively (Garsin et al., 2003; Murakami et al., 2005). In *Drosophila*, disrupting the expression levels of chico, a mammalian IRS homology, resulted in enhanced immune function, improved behavior, and extended lifespan (Martin and Grotewiel, 2006; Libert et al., 2008). Mice lacking IRS-1, although showed mild insulin resistance when young, not only lived longer but also maintained better glucose homoeostasis compared to wild-type controls at older ages (Selman et al., 2009). Consistent with improved metabolic functions, these mice also displayed improved immune profile and motor performance, as well as lowered incidence of osteoporosis, cataract, and ulcerative dermatitis (Selman et al., 2009). Interestingly, mice with a fat-specific insulin receptor knockout show reduced fat mass, alleviated age-related obesity and metabolic abnormalities, and extended lifespan (Bluher et al., 2003; Katic et al., 2007), suggesting a tissue-specific role of the insulin signaling pathway in regulating health span. A correlation relationship between low plasma insulin concentration and reduced mortality risk and lower insulin resistance is also observed in humans (Richardson et al., 2004).

How reducing insulin/IGF-I levels or signaling delays aging-associated diseases and promotes healthy aging? One possible mechanism is through alterations in cell proliferation and apoptosis, which would decrease the incidence of cancer in animals (Hursting et al., 2003). Additionally, reducing insulin/IGF-1 signaling may alter the sensitivity of the animals to oxidative stress and reducing the accumulation of oxidative damage (Richardson et al., 2004; van der Horst and Burgering, 2007). Furthermore, reducing insulin/IGF-1 levels and/or signaling may protect neurons from aging-associated degeneration in the central nervous system (Broughton and Partridge, 2009). Aberrant protein aggregation is a common feature of late-onset neurodegenerative diseases such as Alzheimer’s disease and inhibition of the insulin/IGF signaling pathway has been shown to reduce the toxic aggregate prone proteins in a worm model of Alzheimer’s disease (Cohen et al., 2006; Pinkston-Gosse and Kenyon, 2007). Finally, low insulin/IGF-1 signaling may have anti-aggregation effects that help to maintain cellular protein homeostasis (Morley et al., 2002; Cohen et al., 2006).

A question remains to be answered is how reducing insulin signaling, which is associated with various aging-associated metabolic and cardiovascular diseases such as obesity, type 2 diabetes, and hypertension (Rowe et al., 1983; Kohrt et al., 1993; Finkel and Holbrook, 2000), extends lifespan. Even more puzzling, insulin sensitivity has been shown to be improved by CR and exercise, two types of manipulation that extend longevity (Barger et al., 2003; Teramoto and Bungum, 2010). However, it is interesting to notice that knockout of the insulin receptor in fat tissues increased insulin sensitivity and extend lifespan in mice (Bluher et al., 2003; Katic et al., 2007), suggesting that tissue-specific alteration of the insulin signaling pathway could be the key to promote longevity. Another possible mechanism may be that reducing insulin/IGF-1 signaling leads to down-regulation of certain downstream aging-promoting signaling pathways. Consistent with this, reducing the mTOR signaling pathway, which is known to be activated by insulin and IGF-1 (Astrinidis and Henske, 2005; Taguchi and White, 2008) (Fig. 1), has been shown to improve healthy aging and extend lifespan (Zoncu et al., 2011).

### mTOR signaling: linking energy homeostasis to aging

mTOR is a Ser/Thr protein kinase that integrates signals originating from changes in growth factors, nutrient availability, energy status, and various physiological stresses (Wullschleger et al., 2006; Liu et al., 2009). Convergence of these internal and external signals to the mTOR complex, in turn, triggers various downstream outputs such as mRNA translation, protein synthesis, autophagy, cell proliferation, growth, and survival, which are critical for the lifespan of organisms (Kennedy and Kaeberlein, 2009; McCormick et al., 2011; Zoncu et al., 2011).

mTOR functions in cells by formation of two distinct complexes, mTOR complex 1 (mTORC1) and mTOR complex 2 (mTORC2). These complexes contain unique and shared components and have distinct biological functions in response to nutrients and growth factors (Liu et al., 2009). Both mTORC1 and mTORC2 contain mTOR, mammalian lethal with SEC13 protein 8 (mLST8; also known as GβL), and DEP domain-containing mTOR-interacting protein (DEPTOR). However, mTORC1 contains unique accessory proteins including regulatory-associated protein of mTOR (RAPTOR) and 40 kDa Pro-rich AKT substrate (PRAS40; also known as AKT1S1) whereas mTORC2 contains rapamycin-insensitive companion of mTOR (RICTOR) and other proteins, which distinguish this complex from the mTORC1 (Zoncu et al., 2011).

mTORC1 is rapamycin sensitive and plays a critical role in regulating mRNA translation and protein synthesis in response to nutrients, growth factors, energy, and stress (Zoncu et al., 2011). mTORC1 activity is negatively regulated by the TSC1/2 complex, which inhibits Ras homologue enriched in brain (Rheb), a small guanosine triphosphatase (GTPase) that activates mTOR, via its GTPase-activating protein (GAP) activity (Fig. 1). Growth factors such as insulin and IGF-1 promote AKT-mediated phosphorylation of TSC2, which inhibits the GAP activity of the protein and thus accumulation of Rheb•GTP complex in cells, leading to subsequent activation of mTOR. Phosphorylation of TSC2 by AKT thus provides a direct link between insulin signaling and the nutrient sensor mTOR signaling cascade (Astrinidis and Henske, 2005; Taguchi and White, 2008).

In addition to growth factors, the mTORC1 signaling pathway is also regulated by nutrients such as glucose and amino acids, involving the interaction of mTORC1 with Rag proteins, a different set of small GTPases (Sancak et al., 2008). Very recently, a protein complex named GAP activity toward Rags (GATOR) was identified as a key negative regulator of amino acid-mediated mTORC1 signaling (Bar-Peled et al., 2013; Panchaud et al., 2013). Based on the affinity of protein-protein interactions and their effects on mTORC1, the GATOR proteins can be divided into two sub-complexes, GATOR1 and GATOR2. Inhibition of GATOR1 leads to mTORC1 activation while inhibition of GATOR2 results in mTORC1 inactivation (Bar-Peled et al., 2013). Despite extensive studies on the functional roles of the mTORC1 signaling pathway, only a few direct substrates of mTORC1, including 4E-binding proteins (4E-BPs), 40S ribosomal protein S6 kinases (S6Ks) (Martin and Blenis, 2002; Hay and Sonenberg, 2004), the autophagy inducer ULK1 (Hosokawa et al., 2009; Kim et al., 2011), and PRAS40 (Oshiro et al., 2007; Wang et al., 2008), have been found. Very recently, the growth factor receptor binding protein 10 (Grb10) has been identified as a direct substrate of mTORC1 and phosphorylation of Grb10 by mTORC1 has been suggested to enhance the feedback inhibition of the insulin/IGF-1 signaling pathways (Hsu et al., 2011; Yu et al., 2011) (Fig. 1). However, it is currently unknown whether Grb10 regulates mTORC1 signaling and action *in vivo*.

Studies during the past several years have demonstrated that the mTORC1 signaling pathway contributes to aging and metabolism. Inhibition of the mTORC1 signaling pathway extends lifespan in various model animals, ranging from yeast (Kaeberlein et al., 2005a; Bonawitz et al., 2007; Pan and Shadel, 2009), *C. elegans* (Vellai et al., 2003), fly (Kapahi et al., 2004), to rodents (Harrison et al., 2009; Selman et al., 2009), thus establishing a close relationship between metabolism and aging. Studies from invertebrate models first demonstrated that inhibition of the mTOR signaling pathway is sufficient to reduce protein synthesis and increase lifespan (Vellai et al., 2003; Kapahi et al., 2004; Kaeberlein and Kennedy, 2008; Stanfel et al., 2009). Consistent with these findings, pharmacological inhibition of the mTORC1 signaling pathway with rapamycin confers a robust lifespan extension in genetically heterogeneous mice (Harrison et al., 2009), yeast (Bonawitz et al., 2007), and fruit flies (Kapahi et al., 2004). Inhibition of the mTOC1 signaling pathway has also been shown to inhibit age-related weight gain, decrease aging rate, and delay spontaneous cancer in normal inbred female mice (Anisimov et al., 2011). Taken together, these results support the view that altering metabolism by inhibition of the mTORC1 signaling pathway may be an effective approach for improving health-span and extending lifespan (Kennedy and Kaeberlein, 2009). Consistent with this, mutation of daf-15, the homolog of the mTOR positive regulator RAPTOR in nematodes, led to extended lifespan (Jia et al., 2004). However, the effects of RAPTOR knockout seem to be tissue specific in mice (Polak and Hall, 2009). Adipose-specific RAPTOR knockout mice show similar properties with those long-lived mice, including increased leanness and resistance to diet-induced obesity accompanied by improved glucose tolerance and insulin sensitivity (Polak and Hall, 2009). However, knockout of RAPTOR in skeletal muscle led to muscular dystrophy associated with reduced mitochondrial biogenesis and muscle oxidative capacity but enhanced glycogen storage (Bentzinger et al., 2008). These findings suggest that reduced mTORC1 activity may be beneficial in some tissues while harmful in others. In addition to altering the expression levels of these mTOR regulators, disruption of the expression/activity of mTORC1 substrate S6K has also been shown to extend lifespan in worms (Jia et al., 2004; Hansen et al., 2007), flies (Kapahi et al., 2004), and female mice (Selman et al., 2009). However, whether tissue-specific suppression of S6K has a promoting effect on longevity in higher organisms remains to be established.

The mTORC2 may also be involved in regulation of metabolism and lifespan. On normal diet, mutations of the *C. elegans* homolog of RICTOR, an mTORC2 component, have been shown to increase body fat, slow development, reduce body size, and increase aging rate. However, on nutrient-rich diet, RICTOR mutants showed a profoundly extended life span, which is consistent with decreased consumption of nutrient-rich food by mutants (Soukas et al., 2009). These results indicate that RICTOR plays a critical role in appropriately partitioning calories between long-term energy stores and vital organism processes (Soukas et al., 2009).

Unlike adipose-specific RAPTOR knockout mice, which are resistant to diet-induced obesity (Polak and Hall, 2009), adipose-specific knockout of RICTOR resulted in increased body and organ sizes, independent of dietary fat content (Cybulski et al., 2009). Fat-specific knockout of RICTOR has also been shown to impair insulin-regulated whole body glucose and lipid metabolism (Kumar et al., 2010). However, the effect of fat-specific RICTOR knockout on longevity is currently unclear.

## Mechanisms Underlying The Beneficial Effects Of Suppressing Insulin/Igf-1 and Mtor Signaling On Longevity

**Figure 2:**
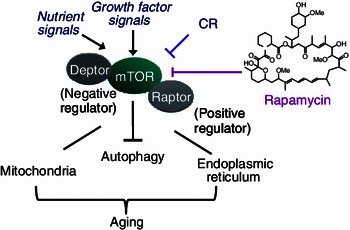
Manipulation of the mTORC1 signaling pathway can promote lifespan extension and healthy aging. As a central energy sensor, the mTORC1 signaling pathway is regulated by various upstream signals such as nutrients, growth factor signaling, and caloric restriction (CR), leading to altered Endoplasmic reticulum (ER) and mitochondrial function and autophagic activity, which play critical roles in aging

Although considerable data have demonstrated that suppression of the insulin/IGF-1 and mTOR signaling pathways are linked to lifespan extension, the underlying mechanisms remain elusive. During the past several years, new evidence begins to emerge on a functional link between these signaling pathways and several key cellular events such as autophagy and the function of ER and/or mitochondria, shedding light on the mechanisms by which suppressing insulin/IGF-1 and mTOR signaling pathways leads to improved longevity and healthy aging (Fig. 2).

### Autophagy: roles in metabolism and aging

Autophagy is a conserved catabolic process that delivers damaged organelles or long-lived proteins to lysosomes for bulk degradation (Kroemer and Levine, 2008; Rubinsztein et al., 2011), which is considered as a mechanism that protects cells against accumulation of damaged organelles and DNA, misfolded proteins, and allows cells to mobilize their energy reserves in response to nutrients depletion, hypoxia, and ER stress (Gonzalez-Polo et al., 2005; Kouroku et al., 2007; Galluzzi et al., 2008; Morselli et al., 2010). During the autophagic process, pre-autophagosome elongates and fuses to form double-membrane vesicle autophagosomes within the cytoplasm. The autophagosomes fuse with acidic lysosomes where the entrapped contents are degraded by proteases. The ER, mitochondria as well as plasma membrane are the major membrane sources contributing to the maturation of pre- or autophagosomal structures (Hayashi-Nishino et al., 2009; Hailey et al., 2010; Ravikumar et al., 2010). More than 30 different genes regulating autophagy have been identified and characterized so far. These autophagy-related genes (Atg) play important roles in the key stages of autophagy process including initiation, elongation, maturation, and fusion with the lysosomes (Ravikumar et al., 2010).

Autophagy is essential for the maintenance of cellular homeostasis and its dysregulation is involved in many metabolic disorders including obesity and insulin resistance, as well as in aging (Meijer and Codogno, 2009; Vellai, 2009; Rubinsztein et al., 2011). Defects in autophagy have been shown to reduce insulin sensitivity in the liver of obese mice with insulin resistance and hyperinsulinemia (Liu et al., 2009; Codogno and Meijer, 2010; Yang et al., 2010). On the contrary, over-expression of *Atg7*, an important gene for autophagy formation, diminished ER stress, improved hepatic insulin sensitivity and fat metabolism, as well as increased peripheral glucose disposal in high fat diet (HFD)-fed or in ob/ob mice (Yang et al., 2010). The suppressive effect of insulin on autophagy in the liver is mediated by FoxO1-mediated transcriptional regulation of *Atg* genes (Liu et al., 2009). In muscle cells, FoxO3 induces expression of a number of autophagy-related genes in response to fasting and denervation (Mammucari et al., 2007; Zhao et al., 2007), and coordinately regulates the activity of both autophagy and the ubiquitin-proteasome associated degradation system in muscle cells (Ravikumar et al., 2010). However, autophagy may action differently in other tissues. In obesity, autophagy has also been found to mediate ER stress-induced reduction of IR and adiponectin in adipose tissues (Chiocchetti et al., 2007; Zhou and Liu, 2010; Zhou et al., 2010). In pancreatic beta cells, autophagy is increased during the initial period of HFD feeding presumably for protecting the beta cells as a compensatory mechanism to boost their insulin production (Ebato et al., 2008). Thus, there may be tissue-specific action of autophagy under the conditions of obesity and insulin resistance.

mTORC1 is an essential negative regulator of autophagy (Takeshige et al., 1992; Ravikumar et al., 2010; Yu et al., 2010). Several mTORC1 target genes, including *Atg13*, *ULK1*, and *ULK2*, are found to be involved in the initiation step of autophagosome formation in mammalian cells (Kim et al., 2011; Shang and Wang, 2011). Atg13 and ULK1/2 interact with FIP200 to form a ULK1/2-Atg13-FIP200 stable complex that signals to the autophagic machinery downstream of mTOR (Hay and Sonenberg, 2004; Ganley et al., 2009; Hosokawa et al., 2009). Under nutrient-rich conditions, mTORC1 suppresses autophagy through direct interaction with this complex, which leads to phosphorylation-dependent inhibition of Atg13 and ULK1 (Ravikumar et al., 2010; Shang and Wang, 2011). Under the conditions of starvation or rapamycin treatment, however, mTORC1 dissociates from the complex, leading to dephosphorylation-dependent activation of ULK1 and ULK2, which finally triggers autophagy (Ganley et al., 2009; Hosokawa et al., 2009; Kamada et al., 2010). On the other hand, however, mTOR may be feedback-regulated by autophagy. Overexpression of Atg1 inhibits TOR signaling in *Drosophila*, presumably as a negative feedback on the activity of TOR and to further enhance induction of autophagy (Scott et al., 2007).

Accumulating evidence suggests that autophagy-associated signaling and genes are involved in cell survival, cell death, and aging (Meijer and Codogno, 2009; Vellai, 2009). It has been observed that autophagic activity declines with age and this decline is associated with accumulation of damaged proteins and organelles, a common characteristic feature of aging (Vittorini et al., 1999; Cuervo and Dice, 2000). Up-regulation of important components of the autophagy process prevents age-dependent neuronal damage and enhances longevity in *Drosophila* (Simonsen et al., 2008). Mutation of autophagy-related gene *Atg7* results in hypersensitive to nutrient and oxidative stress and decreased lifespan in *Drosophila* (Juhász et al., 2007). In addition, induction of autophagy by pharmacological reagents such as rapamycin, resveratrol or the natural polyamine spermidine, caloric restriction, or genetic manipulations such as knocking down the autophagy inhibitor p53, all have been shown to improve animal survival and reduce age-related mortality in *C. elegans* (Jia et al., 2004; Tavernarakis et al., 2008). Recent studies indicate that autophagy may modulate aging in germline-less *C. elegans* through coordination with lipid metabolism to prolong life span in a mTOR-dependent manner (Lapierre et al., 2011, 2012). Furthermore, inhibition of autophagy by knocking-out or knocking-down essential *Atg* genes leads to apoptosis or necrosis and prevents the longevity promoting effect of CR (Meléndez et al., 2003; Boya et al., 2005; Madeo et al., 2010).

An interesting question remains to be fully addressed is whether activation of autophagy mediates the beneficial effects of suppressing insulin/IGF-1 or mTOR signaling on longevity. The studies in various invertebrate animal models demonstrate that autophagy may be involved in mTOR signaling-associated lifespan regulation. Earlier studies show that autophagy is essential for dauer development and lifespan extension in *C. elegans* (Meléndez et al., 2003). The mutation of worm bec-1 (Beclin 1), homologue of yeast VPS30/mammalian beclin1, as well as atg-7 and atg-12, blocks lifespan extension by a daf-2 mutant (Clancy et al., 2001; Meléndez et al., 2003). Indeed, autophagy activation has been found to be a common feature of all the long-lived mutant worms (Hansen et al., 2008; Toth et al., 2008). In yeast, autophagy is required for normal survival as well as lifespan extension by rapamycin (Alvers et al., 2009a, 2009b). Similar results were found in *Drosophila*, in which inhibition of autophagy abrogates rapaymcin-dependent lifespan extension (Bjedov et al., 2010). Furthermore, studies in fly and mouse models of Huntington disease discovered that inhibition of mTOR induces autophagy and reduces toxicity of polyglutamine expansions and aggregate formation (Ravikumar et al., 2004). However, whether autophagy plays similar roles in decreased metabolic signaling associated lifespan extension in vertebrates still needs to be clarified.

CR is the most efficient inducer of autophagy and inhibition of autophagy diminishes the anti-aging effects of CR in all species investigated (Levine and Kroemer, 2008; Rubinsztein et al., 2011). CR-induced autophagy may be mediated by activation of either AMP-activated protein kinase (AMPK) or Sirtuin 1 (SIRT1) (Cantó et al., 2010; Morselli et al., 2010), two important cellular energy sensors. Moreover, CR can induce autophagy through the inhibition of insulin/IGF-1 and mTOR signaling pathways (Kenyon, 2010).

### ER stress: role in insulin/IGF-1 and mTOR signaling and aging

ER plays critical roles in protein translation, folding, modification, and transportation to its final cellular destination. Under pathophysiological conditions in which increased misfolded or mutant proteins accumulated in the lumen, ER initiates an adaptive stress response pathway known as unfolded protein response (UPR) to reestablish protein equilibrium (Harding and Ron, 2002; Ron and Walter, 2007; Yoshida, 2007; Ron and Hubbard, 2008).

ER stress was first identified as a response to glucose limitation, which leads to accumulation of misfolded proteins in the ER due to impaired protein glycosylation. It was later shown that UPR could be activated in response to both glucose and oxygen deprivation thus functions as a sensor for cell energy status (Kauffman et al., 2002). Numerous studies have demonstrated that ER stress plays a critical role in the progress of chronic metabolic diseases such as obesity, insulin resistance, and type 2 diabetes (Harding and Ron, 2002; Fonseca et al., 2007; Hotamisligil, 2010), as well as atherosclerosis (Vasa-Nicotera, 2004; Zhao and Ackerman, 2006) and the aging-related neurodegenerative diseases (Lindholm et al., 2006; Yoshida, 2007). Thus, ER stress response and related signaling networks are emerging as potential intersection sites of metabolic disease and longevity.

Aging is associated with a decline in the expression and activity of several key molecular chaperones and folding enzymes responsible for proper protein folding and the adaptive response of the UPR, including immunoglobulin heavy chain-binding protein (BiP)/glucose regulated proteins 78 (GRP78), calnexin, calreticulin, and protein disulfide isomerase (PDI) (Naidoo, 2009), which may partly attribute to age-associated increase in protein misfolding and aggregation. However, some other ER stress related proteins such as the pro-apoptotic marker CCAAT/enhancer-binding protein-homologous protein (CHOP) and ER induced apoptosis marker caspase-12 are increased during aging. CHOP levels are elevated in the brain and other tissues of aged mice (Paz Gavilan et al., 2006; Naidoo et al., 2008) and in aged animals with sleep deprivation-induced ER stress (Naidoo et al., 2008). It is well established that CHOP mediates apoptosis in response to ER stress (Wang et al., 1996; Zinszner et al., 1998; Tabas and Ron, 2011). Elevated CHOP levels have also been shown to sensitize cells to oxidative insults (Ikeyama et al., 2003) and increase ROS levels in rat fibroblasts (McCullough et al., 2001).

Information on the link between insulin/IGF-1 or mTOR signaling and ER stress in life span determination remains limited. However, available evidence suggests a close link between these metabolic signaling pathways and ER, which makes it a potential anti-aging target. Induction of ER stress by chemicals or obesity has been suggested as a key mechanism leading to insulin resistance and type 2 diabetes (Ozcan et al., 2004, 2006). Under the condition of ER stress, activated c-Jun N-terminal kinase (JNK) inhibits insulin signaling through phosphorylation of IRS-1 at serine 307, leading to the development of insulin resistance (Aguirre et al., 2000; Hirosumi et al., 2002). Recent studies in *C. elegans* show that a decrease in the expression levels of inositol requiring enzyme 1 (IRE1) and X-box binding protein 1 (XBP-1), two key molecules involved ER-associated degradation (ERAD), shorten lifespan extension induced by insulin/IGF-1 signaling mutation (Henis-Korenblit et al., 2010). Notably, XBP-1 has been shown to function synergistically with DAF-16 to activate *dox-1*, a newly identified longevity gene, leading to enhanced resistance to ER stress and extended life span in *daf-2* mutants (Henis-Korenblit et al., 2010). However, the underlying mechanisms remain elusive. It is also unclear whether the IRE-1/XBP-1 axis is altered during aging, which would impede the coordination with other stress response factors and consequently impair the ER stress response.

Emerging data indicate that ER is closely associated with the autophagic process. ER is one of the major membrane sources contributing to the maturation of pre- or autophagosomal structures and thus structurally connected to the autophagic machinery (Hayashi-Nishino et al., 2009; Hailey et al., 2010). In addition to the structural connection, ER also functions closely with autophagy under both physiological and pathophysiological conditions. It has been shown that ER stress induces autophagy in mammalian cells (Ogata et al., 2006), which provides an alternative mechanism to remove misfolded proteins that cannot be degraded by ERAD and thus assists ER homeostasis and cell survival (Ding and Yin, 2008). Phosphorylation of PKR-like eukaryotic initiation factor 2α kinase (PERK) and eukaryote initiation factor 2α (eIF2), two molecules important for translational regulation and cell survival during ER stress, has been shown to be essential for autophagy formation (Kouroku et al., 2007). As a result, defected autophagy in liver leads to ER stress and insulin resistance in obesity (Codogno and Meijer, 2010; Yang et al., 2010).

Although both mTOR and ER stress signaling have attracted wide attention in fundamental cell biology and drug discovery, evidence on the crosstalk between the two pathways has emerged only very recently (Appenzeller-Herzog and Hall, 2012). As a key regulator of protein synthesis, mTORC1 controls both upstream and downstream of ER stress signals. Conversely, ER stress is able to activate mTORC1 via ATF6a, which triggers the PI3K pathway and increases the levels of RHEB by unknown mechanisms (Appenzeller-Herzog and Hall, 2012). Chronic ER stress leads to phosphorylation of the mTORC2 component RICTOR by GSK3b, resulting in suppression of Akt activation and glucose metabolism (Chen et al., 2011).

The interplay between mTOR signaling and UPR is particularly important in stress-induced apoptosis. Under unfavorable growth conditions, activation of TSC1/TSC2 would inhibit cell growth and thus protects cells from the harmful environment. While under the conditions of TSC mutation or rich in nutrients, mTORC1 is constitutively activated to stimulate translation and promotes cell growth, which has been found to cause ER stress (Ozcan et al., 2004; Kang et al., 2011). Cells with mutation in either TSC1 or TSC2 are hypersensitive to ER stress and undergo apoptosis. In addition, defects in ER stress response in TSC mutant cells could be restored by RAPTOR knockdown or by RHEB activation (Kang et al., 2011), demonstrating a functional link between mTOR signaling and ER stress. Consistent with this, a recent study found that ER stress robustly activated mTORC1, which in turn induced apoptosis (Kato et al., 2012). However, while these results demonstrate a positive relationship between ER stress and mTOR in apoptosis, other studies suggest a negative correlation between ER stress and mTOR. TSC-deficient cells have been found to be more resistant to ER stress-induced autophagy, probably due to constitutive activation of mTOR (Qin et al., 2010). In response to oxidative and ER stress, activating transcription factor 4 (ATF4) and CCAAT/enhancer-binding protein-beta (C/EBP-beta) negatively regulate mTOR by stimulating the expression of Redd1 (Jin et al., 2009), a known inhibitor of mTOR (Corradetti et al., 2005) whose expression is induced by a variety of cellular stress conditions, including hypoxia and energy stress (Sofer et al., 2005).

Although direct evidence remains limited, some indirect evidence postulates a potential linkage between ER stress and mTOR in lifespan determination. Studies in worms show that some genes that are involved in ER stress mediate lifespan extension and TOR signaling (Jia et al., 2004; Steffen et al., 2008). For example, GCN4, a nutrient-responsive transcription factor that regulates diverse cellular processes including autophagy and ER stress response (Natarajan et al., 2001; Patil et al., 2004), has been found to mediate lifespan extension in yeast (Steffen et al., 2008). In addition, the hypoxia inducible factor-1 (HIF-1), one of the targets of the mTOR pathway in mammalian cells, has been shown to be involved in CR-induced lifespan extension in *C. elegans* (Jia et al., 2004).

### Mitochondria: the cellular powerhouse and a primary determinant of longevity

Mitochondrion is another important player that may mediate insulin/IGF-1 or mTOR signaling in aging. As major energy-generating organelles in eukaryotic cells, mitochondria are essential in maintaining cellular energy supplies by generating adenosine triphosphate (ATP) through oxidative phosphorylation (OXPHOS). Mitochondria are also one of the primary sites for the production of reactive oxygen species (ROS), which are generated as a toxic by-product during OXPHOS. Various studies have demonstrated that increased ROS and oxidative stress as one of the important causes of mammalian aging (Harman, 1956; Finkel and Holbrook, 2000; Wallace, 2005).

It is well established that mitochondrial function and activity are primary determinants of longevity. Defects in mitochondrial function and/or reduction in mitochondrial numbers are closely associated with many age-related diseases, including metabolic syndrome, neurodegenerative diseases, and cancer (Wallace, 2005). A progressive loss of mitochondrial energetic capacity, which is observed in diverse organisms including humans, is linked to age-associated decline in the expression of genes important for mitochondrial electron transport chain (ETC) function and energy metabolism (Petersen et al., 2003; McCarroll et al., 2004; Zahn et al., 2006).

Insulin/IGF-1 signaling has been shown to regulate mitochondrial DNA and OXPHOS protein syntheses, oxidative capacity, and ATP production and dysregulation in insulin signaling is associated with mitochondrial dysfunction that leads to various metabolic diseases (Kelley et al., 2002; Lowell and Shulman, 2005), although the causal relationship between insulin resistance and mitochondrial dysfunction remains to be further defined (Turner and Heilbronn, 2008). It is still puzzling as to whether and how suppressed insulin/IGF-1 signaling is associated with improved mitochondrial function in longevity. Studies in a mouse model of Huntington’s disease show that reducing IRS2 level in brain induces lifespan extension of animals with improved mitochondrial function, autophagy, and oxidative stress resistance (Sadagurski et al., 2011), which is linked to increased nuclear localization of the transcription factor FOXO1 and expression of FOXO1-dependent genes that exert these beneficial effects. However, another study found that knockout of both Irs-1 and Irs-2 in the liver activated the FOXO1 target gene Hmox1 (heme oxygenase-1), leading to disruption of complex III and IV of the respiratory chain, lower NAD^+^/NADH ratio, and reduced ATP production (Cheng et al., 2009). Apparently, there is a tissue-specific regulation of mitochondrial activity by insulin in aging.

The mTOR signaling has been shown as a direct regulator of mitochondrial function (Ramanathan and Schreiber, 2009). In skeletal muscle, inhibition of mTOR by rapamycin decreases the gene expression of peroxisome proliferator-activated receptor γ (PPARγ) coactivator 1α (PGC-1α), a master regulator of mitochondrial biogenesis in many tissues, and estrogen-related receptor α and nuclear respiratory factors, resulting in a decrease in mitochondrial gene expression and oxygen consumption (Schieke et al., 2006; Narasimhan et al., 2009; Hwang et al., 2012), suggesting a positive role of mTOR in mitochondrial function.

Interestingly, a series of studies conducted in yeast showed that inhibition of TOR signaling extended life span via modulation of mitochondrial respiration, function, and gene expression. For example, deletion of the RAPTOR extended lifespan in yeast by increasing mitochondrial respiration via enhanced translation of mtDNA-encoded oxidative phosphorylation complex subunits (Bonawitz et al., 2007). Reducing TORC1 signaling by rapamycin couples respiration and ROS during growth, which extends chronological lifespan in yeast (Pan et al., 2011).

Accumulation of damaged proteins in mitochondria is a feature common to all aged cells. Mitochondria damage-induced autophagy or “mitophagy” is one of the major degradation pathways in mitochondrial turnover and homeostasis (Kim et al., 2007). Age-associated loss of autophagy leads to accumulation of damaged mitochondria, which triggers cell death and inflammation (Green et al., 2011). Mitophagy can be induced by nutrient deprivation, oxidative stress, hypoxia, mitochondrial dysfunction, and alterations of mitochondrial permeability transition (MPT) (Lemasters, 2005). Elimination of damaged mitochondria by autophagy serves as a rescue mechanism for cells to escape from cell death (Codogno and Meijer, 2005). Recent studies show that targeted mitochondrial damage led to up-regulation of autophagy genes *LC3B*, *ATG5,* and *ATG12* in human endothelial cells (Mai et al., 2012). On the other hand, over-expression of these genes improved mitochondrial membrane potential and enhanced ATP production, which might contribute to increased cellular longevity (Mai et al., 2012).

Some major neurodegenerative diseases, including Parkinson’s disease (PD), Huntington’s disease, and Alzheimer’s disease, are linked to defects in mitochondria and autophagy. PD is caused by the selective loss of dopaminergic neurons, which can be induced by mitochondrial toxins. Several genes, inlcuding PINK1 (phosphatase and tensin homolog (PTEN)-induced putative kinase 1), Parkin, or DJ1, have been shown to play important roles in mitophagy and mutations of these genes often link to autosomal recessive PD (Geisler et al., 2010; Youle and Narendra, 2011). As a critical determinant of mitophagy, Parkin is selectively recruited to mitochondria with low membrane potential, which facilitates the engulfment of mitochondria by autophagosomes (Narendra et al., 2008). Overexpression of Parkin eliminates mitochondria with deleterious mutations in cytochrome oxidase subunit I (COXI) (Suen et al., 2010), suggesting that Parkin may mediate a mitochondrial quality control pathway to maintain organelle homeostasis. There is some evidence suggesting that mTOR may be involved in Parkin relocation and mitophagy (Gilkerson et al., 2011). However, the molecular mechanisms underlying the cross-talk between mitochondrial dysfunction and autophagy remain to be further elucidated.

## Future Directions and Concluding Remarks

Recent advances have significantly increased our understanding of how aging and health-span are regulated by metabolic signals in response to cellular, nutrient, and environmental cues. An emerging view is that aging and metabolic dysfunction are closely associated and interventions that improve metabolism may also extend lifespan. Several manipulations, such as suppressing the insulin/IGF-1 and the mTOR signaling pathways, improving ER and mitochondrial function, and activating the autophagic process, have been shown to effectively slow down the aging process in various species ranging from yeast, flies to rodents. However, while these findings raise great hope to improve health-span and extend lifespan by targeting metabolic signaling pathways, many issues remain to be resolved before these interventions can be effectively applied to clinic. One of the major challenges is that these metabolic signaling pathways have tissue-specific function and whole-body suppressing these pathways may lead to metabolic disorders and accelerated aging. Thus, developing therapeutic drugs targeting tissue-specific signaling pathways would be of high clinic value. One of the promising targets is adipose insulin signaling pathway. Consistent with this, fat-specific disruption of this signaling pathway led to extended lifespan and improved metabolism (Bluher et al., 2003; Katic et al., 2007). Another possible target for aging is the mTORC1 signaling pathway in adipose tissues. Suppressing insulin and mTOR signaling pathways in adipose tissues may promote the biosynthesis and secretion of adipokines that are beneficial for health. Fat-specific suppression of these signaling pathways, on the other hand, may decrease biosynthesis and secretion of adipokines that are harmful to metabolism and longevity. Consistently, significantly higher levels of adiponectin, an anti-inflammatory and insulin sensitizing adipokine, have been found in centenarians (Bik et al., 2006).

Due to the complex nature of aging and metabolism, it is not yet clear whether targeting key signaling pathways involved in metabolism in specific tissues will actually improve healthy aging in humans. It is also unclear how tissue-specific signaling pathways could be effectively and specifically targeted. New strategies to suppress tissue-specific insulin and/or the mTOR signaling pathways or to mimic CR response in humans should be of significant value to improve metabolism and extend health-span. Further studies are thus warranted to elucidate the tissue-specific signaling pathways involved in the regulation of metabolism and aging, which is essential for the development of effective small compounds that exert tissue-specific function on healthy lifespan.

## Abbreviations

4E-BPs: 4E-binding proteins
AMPK: AMP-activated protein kinase
ATF4: activating transcription factor 4
Atg: autophagy-related genes
ATP: adenosine triphosphate
Beclin 1: bec-1
BiP/GRP78: binding protein/glucose regulated proteins 78
C/EBP-beta: CCAAT/enhancer-binding protein-beta
CHOP: CCAAT/enhancer-binding protein-homologous protein
COXI: cytochrome oxidase subunit I
CR: caloric restriction
DEPTOR: DEP domain-containing mTOR-interacting protein
DKO: double-knockout
eIF2: eukaryote initiation factor 2α
ER: endoplasmic reticulum
ERAD: ER-associated degradation
ETC: electron transport chain
FOXO: forkhead box O
GAP: guanosine triphosphatase-associated protein
Grb10: growth factor receptor binding protein 10
HFD: high fat diet
HIF-1: hypoxia inducible factor-1
Hmox1: heme oxygenase-1
IGF-1: insulin-like growth factor 1
IR: insulin receptor
IRE1: inositol requiring enzyme 1
IRS: insulin receptor substrate
JNK: c-Jun N-terminal kinase
mLST8: mammalian lethal with SEC13 protein 8
MPT: mitochondrial permeability transition
mTOR: mammalian target of rapamycin
mTORC1: mTOR complex 1
mTORC2: mTOR complex 2
OXPHOS: oxidative phosphorylation
PD: Parkinson’s disease
PDI: protein disulfide isomerase
PDK1: 3-phosphoinositide-dependent protein kinase 1
PGC-1α: peroxisome proliferator-activated receptor γ coactivator 1α
PI3K: phosphoinositide 3-kinase
PINK1: phosphatase and tensin homolog-induced putative kinase 1
PPARγ: peroxisome proliferator-activated receptor γ
PRAS40: 40 kDa Pro-rich AKT substrate
PTEN: phosphatase and tensin homolog
RAPTOR: regulatory-associated protein of mTOR
RHEB: Ras homologue enriched in brain
RICTOR: rapamycin-insensitive companion of mTOR
ROS: reactive oxygen species
S6Ks: S6 kinases
SIRT1: Sirtuin 1
TSC2: tuberous sclerosis 2
UPR: unfolded protein response
XBP-1: X-box-binding protein-1
